# Association of mast cells with lung function in chronic obstructive pulmonary disease

**DOI:** 10.1186/1465-9921-9-64

**Published:** 2008-09-10

**Authors:** Margot ME Gosman, Dirkje S Postma, Judith M Vonk, Bea Rutgers, Monique Lodewijk, Mieke Smith, Marjan A Luinge, Nick HT ten Hacken, Wim Timens

**Affiliations:** 1 Department of Pulmonology, University Medical Center Groningen and University of Groningen, Groningen, The Netherlands; 2 Department of Epidemiology, University Medical Center Groningen and University of Groningen, Groningen, The Netherlands; 3 Department of Pathology, University Medical Center Groningen and University of Groningen, Groningen, The Netherlands

## Abstract

**Background:**

In asthma, higher chymase positive mast cell (MC-C) numbers are associated with less airway obstruction. In COPD, the distribution of MC-C and tryptase positive mast cells (MC-T) in central and peripheral airways, and their relation with lung function, is unknown. We compared MC-T and MC-C distributions in COPD and controls without airflow limitation, and determined their relation with lung function.

**Methods:**

Lung tissue sections from 19 COPD patients (median [interquartile range] FEV_1_% predicted 56 [23–75]) and 10 controls were stained for tryptase and chymase. Numbers of MC-T and MC-C were determined in different regions of central and peripheral airways and percentage of degranulation was determined.

**Results:**

COPD patients had lower MC-T numbers in the subepithelial area of central airways than controls. In COPD, MC-T numbers in the airway wall and more specifically in the epithelium and subepithelial area of peripheral airways correlated positively with FEV_1_/VC (Spearman's rho (r_s_) 0.47, p = 0.05 and r_s _0.48, p = 0.05, respectively); MC-C numbers in airway smooth muscle of peripheral airways correlated positively with FEV_1_% predicted (r_s _0.57, p = 0.02). Both in COPD patients and controls the percentage of degranulated MC-T and MC-C mast cells was higher in peripheral than in central airways (all p < 0.05), but this was not different between the groups.

**Conclusion:**

More MC-T and MC-C in peripheral airways correlate with better lung function in COPD patients. It is yet to determine whether this reflects a protective association of mast cells with COPD pathogenesis, or that other explanations are to be considered.

## Introduction

Chronic obstructive pulmonary disease (COPD) is characterized by inflammation in both central and peripheral airways [1], which is dominated by neutrophils, macrophages, T lymphocytes (mainly CD8^+ ^cells), and B lymphocytes [2-4]. In addition, some [5,6] but not all studies [7,8], have demonstrated higher numbers of mast cells in patients with COPD than in controls without airflow limitation. Theoretically, mast cells could play a role in the pathogenesis of COPD [9] by inducing collagen production [10] fibroblast proliferation [11,12], and release of various mediators, including the potent proteases tryptase and chymase. Tryptase, a mast cell specific protease, is present in all human mast cells, whereas chymase is present in a subset of mast cells.

Balzar and co-workers recently evaluated the number of chymase and tryptase positive mast cells in central and peripheral airways of patients with severe asthma [13]. They demonstrated that higher numbers of chymase positive mast cells in peripheral airways are associated with less severe airflow limitation. Therefore, they suggested that mast cells are protective for enhanced airway obstruction in patients with asthma.

To our knowledge, the distribution of chymase and tryptase positive mast cells in central and peripheral airways, and their relation with lung function has not been investigated previously in patients with COPD. This study did so and compared this distribution with results in controls without airflow limitation.

## Methods

### Subjects and tissue collection

Tissue samples were collected from 29 individuals: 19 with COPD and 10 controls without airflow limitation (Table 1). COPD patients had no chronic bronchitis or α_1_-antitrypsin deficiency (10 males, median [interquartile range] age 62 [54–72], pack-years 31 [27–40]) and they were former- or current smokers (14 and 5 patients, respectively). They had mild to very severe COPD according to the Global Initiative on Lung Disease (GOLD) criteria (stage I, II, III, IV in 3, 8, 1, and 7 patients, respectively) [14]. Forced expiratory volume in one second (FEV_1_) as percentage of predicted (% pred) was 56 [23–75], with an FEV_1_/vital capacity (VC) ratio of 50% [30–59]. Thirteen out of 19 patients with COPD were treated with oral or inhaled corticosteroids or their combination. Controls without airflow limitation and without corticosteroid use (8 males, age 56 [47–70], pack years 30 [25–50]) were former (n = 5) or current (n = 4) smokers; smoking status of 1 control was unknown. Median [range] FEV_1_% pred of the control group was 103 [90–106], and FEV_1_/VC ratio 72% [72–75]. Lung function data were missing from one control, a 43-year-old male organ donor, from whom we obtained lung tissue that had not been used for transplantation because of logistic (unilateral transplantation) reasons. An experienced pulmonary pathologist (WT) found no signs of COPD or other significant pathology in lung tissue.

**Table 1:** Characteristics of COPD patients and controls

	**Controls**	**COPD**
N	10	19
Male/female	8/2	10/9
Current smoker (y/n/unknown)	4/5/1*	5/14/0
Age (median IQR)	56 (47–70)	62 (54–72)
Packyears (median (range))	30 (25–50) *	31 (27–40)
ICS (y/n/unknown)	0/9/1*	8/11/0
OCS (y/n/unknown)	0/9/1*	7/12/0
ICS and/or OCS (y/n/unknown)	0/9/1*	13/6/0
B2 agonist (y/n/unknown)	0/9/1*	10/9/0
Anticholinergics (y/n/unknown)	0/9/1*	10/9/0
COPD Severity Stage (GOLD 1/2/3/4)	-	3/8/1/7
FEV_1 _(% predicted)	103 (92–107)**	56 (23–75)
FEV_1_/IVC (%)	72 (72–75)**	50 (30–59)

Data are presented as median (IQR)

* n = 9; 1 subject (anonymous donor) with some missing data; ** n = 8, 2 subjects (incl. donor) with missing lung function data

Lung tissue samples from GOLD stage IV COPD patients were obtained from tissue remaining after standard pathology protocols in case of lung transplant procedures. The other tissue samples were obtained from subjects undergoing partial resection, (bi)lobectomy, or pneumonectomy for pulmonary carcinoma or metastasis. None of the patients had received chemotherapy. Lung tissue was taken as distant as possible from the tumor, or from non-involved lobes. The study protocol was consistent with national ethical and professional guidelines ("Code of Conduct; Dutch Federation of Biomedical Scientific Societies"; ).

### Tissue processing/immunohistochemistry

**Figure 1:**
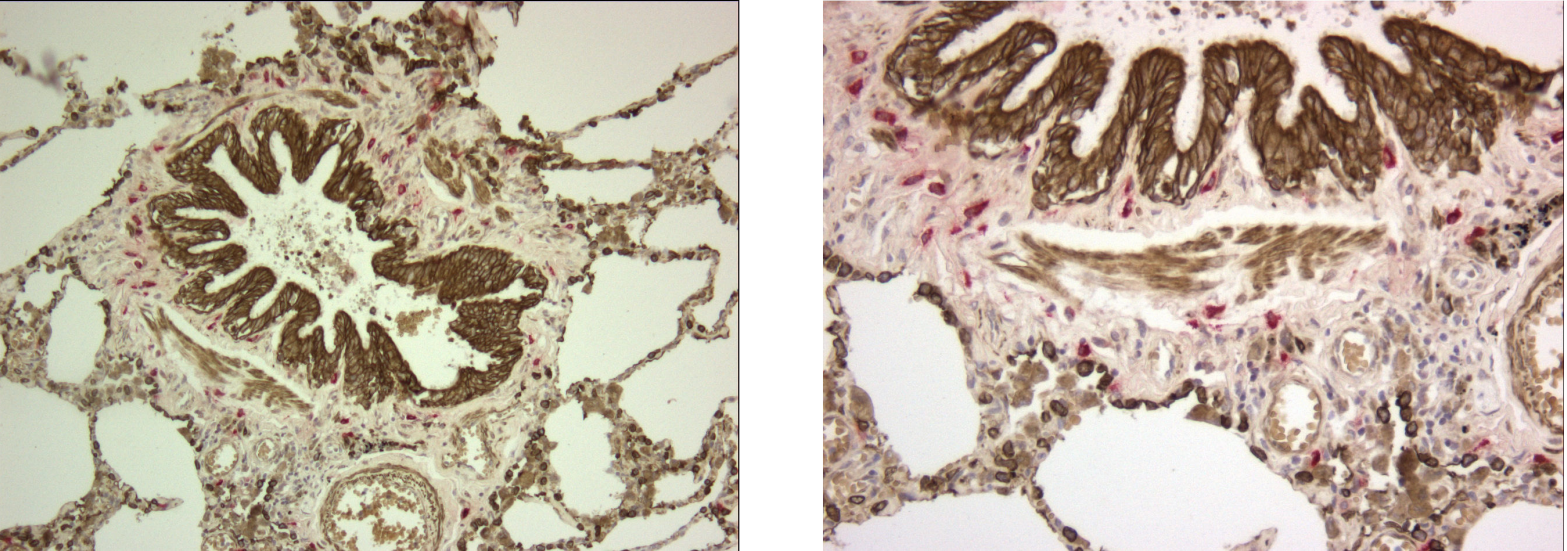
1a. (left) Double immunostaining: tryptase positive mast cells (bright red) in a small airway in a tissue sample from a patient with moderate COPD (GOLD stage 2); bronchial and alveolar type 2 epithelial cells and smooth muscle stain golden brown, magnification × 100; 1b. (right) larger magnification (× 200).

Immunohistochemistry was performed on 3 μm formalin fixed, paraffin embedded lung tissue sections. Consecutive tissue sections were stained for tryptase (AA1, DAKO, Glostrup, Denmark) and chymase (CC1, Abcam, Cambridge, UK) using an immuno-alkaline phosphatase method with goat-anti-mouse immunoglobulin conjugated to alkaline phosphatase as a second step and Fast Red/Napthol AS-Mx as a chromogen providing a bright red reaction product. Double staining was performed to delineate the different compartments. After chymase or tryptase staining, slides were incubated with a mixture of mouse monoclonal antibodies detecting anti-alpha-smooth-muscle actin (Progen, Heidelberg, Germany; clone ASM-1) and pan-keratin (AE1-AE3, Boehringer Mannheim, Mannheim, Germany). As a detection step, slides were incubated with rabbit anti-mouse immunoglobulin conjugated to peroxidase. 3'3'di-amino-benzidin together with H_2_O_2 _was used as a chromogen, providing a gold-brown reaction product. This double staining procedure with subsequent incubation steps allowed easy identification of mast cells with clear demarcation of smooth muscle area and delineation of bronchial epithelium, as well as outer limit of adventitia by (mainly type 2) alveolar epithelial cells (see figure 1 for illustration). Appropriate isotype matched control sera were used for negative controls.

### Definition of lung regions and morphometric analysis

We examined 1 central airway and, depending on the availability, 1 to 5 peripheral airways from each subject. Central airways were defined as cartilaginous airways, maximum from the third generation. Three central airway regions were analyzed separately: 1) epithelium; 2) subepithelial area (area between the bronchial epithelium and smooth muscle layer); and 3) airway smooth muscle.

Peripheral airways were defined as airways with a circumferential size of maximally 6 mm, and a variation of maximal versus minimal diameter ≤ two-fold. Four peripheral airway regions were analyzed separately: 1) epithelium; 2) subepithelial area; 3) airway smooth muscle; and 4) adventitia (area outside smooth muscle to the connection points of the surrounding parenchyma).

All areas were measured using the Leica QuantiMed morphometric system (Leica Microsystems, Lübeck, Germany) and chymase and tryptase positive, nucleated cells were counted manually within each area and in addition related to all these areas together.

The percentage of degranulation was determined for all MC-C and MC-T mast cells, using the method of Carroll et al [15]. In short, at a high power (x400) magnification, positively-stained nucleated mast cells were classified as intact if they were dense, compact, had unbroken cytoplasmic boundaries and did not have any surrounding positively stained granules. All other nucleated tryptase or chymase positive cells were classified as degranulated. Degranulated cells were expressed as a percentage of the total number of nucleated MC-T or MC-C-positive cells for the whole small or large airway section.

A random selection of 10% of all tryptase sections was recounted to determine the intra-class correlation coefficient (ICC), reflecting level of agreement for repeat counts by the same observer. The same was done by two independent observers to determine inter-observer agreement.

### Statistical analysis

Means and standard deviations or medians with interquartile ranges (IQR) of variables were calculated. Numbers of chymase and tryptase positive mast cells were expressed per mm length of basement membrane (epithelium) or mm^2 ^area (subepithelial area, airway smooth muscle, and adventitia). In case two or more peripheral airways from one subject were examined, mean cell concentration of all peripheral airways from this subject was used for statistical analysis. Differences between groups were analyzed using Chi-Square or Mann-Whitney U-tests, paired analyses within groups using Wilcoxon-rank-sum-tests, and correlations between cell concentrations and lung function using Spearman's rank correlation coefficient (r_s_). SPSS 12.0 (SPSS Inc., Chicago, IL) software was used for statistical analysis. A p-value of 0.05 or less was considered significant.

## Results

### Patient characteristics (Table 1)

Patients with COPD had significantly lower FEV_1 _(% pred) and FEV_1_/VC values than controls without airflow limitation (both p < 0.001). Gender, smoking status, and pack-years smoking were similar in the two groups (p > 0.05).

### Morphometric analysis

#### Central airways

Median [IQR] length of epithelium analyzed in the total study population was 7.5 mm [7.3–7.6], median tissue area 0.7 mm^2 ^[0.5–1.2] for the subepithelial area, and 0.6 mm^2 ^[0.4–0.9] for airway smooth muscle.

#### Peripheral airways

The length of epithelium per tissue section analyzed in the total study population was 2.3 mm [2.0–2.8] per peripheral airway. Median tissue area analyzed was 0.02 mm^2 ^[0.02–0.04] for the subepithelial area, 0.02 mm^2 ^[0.01–0.03] for airway smooth muscle, and 0.1 mm^2 ^[0.1–0.2] for the adventitia. Comparable tissue areas were analyzed in the two groups (p > 0.05).

### Distribution of mast cells in central and peripheral airways in COPD and controls (Table 2, Figure 1))

**Table 2:** 

**a**. The concentration of tryptase positive mast cells (MC-T) per region for patients with COPD and controls		
	**Controls** **(n = 10)**	**COPD** **(n = 19)**

***Central airways***		
Epithelium (cells/mm)	0.07 [0.0–0.9]	0.0 [0.0–0.1]
Subepithelial area (cells/mm^2^)	108.8 [83.9–190.2]	53.7 [25.7–120.8]^¶^
Airway smooth muscle (cells/mm^2^)	32.5 [15.8–49.9]	20.1 [10.5–33.3]
% degranulated mast cells	35.9 [21.9–51.2]	28.0 [22.4–37.4]
***Peripheral airways***		
Epithelium (cells/mm)	0.0 [0.0–0.9]	0.0 [0.0–0.2]
Subepithelial area (cells/mm^2^)	247.9 [73.9–548.2]	184.6 [100.4–282.3]
Airway smooth muscle (cells/mm^2^)	12.6 [0.0–36.9]	18.4 [0.0–47.6]
Adventitia (cells/mm^2^)	279.4 [79.2–646.9]	214.9 [107.2–327.2]
% degranulated mast cells	68.6 [41.5–81.4]	67.5 [53.0–84.1]

**b**. The concentration of chymase positive mast cells (MC-C) per region for patients with COPD and controls		

	**Controls** (n = 10)	**COPD** (n = 19)

***Central airway samples***		
Epithelium (cells/mm)	0.0 [0.0–0.0]	0.0 [0.0–0.3]^¶¶^
Subepithelial area (cells/mm^2^)	18.1 [8.2–28.9]	11.0 [4.5–28.3]
Airway smooth muscle (cells/mm^2^)	10.6 [2.7–15.2]	3.5 [0.0–6.0]
% degranulated mast cells	33.3 [25.7–52.2]	40.0 [26.8–58.3]
***Peripheral airways***		
Epithelium (cells/mm)	0.0 [0.0–0.0]	0.0 [0.0–0.2]
Subepithelial area (cells/mm^2^)	19.4 [0.0–71.8]	54.5 [0.0–160.0]
Airway smooth muscle (cells/mm^2^)	0.0 [0.0–54.1]	0.0 [0.0–50.7]
Adventitial layer (cells/mm^2^)	128.0 [44.1–184.0]	68.5 [13.2–162.7]
% degranulated mast cells	66.8 [55.6–93.2]	63.9 [52.1–95.0]

Data are presented as median [interquartile range]; ^¶ ^p = 0.035 versus controls; ^¶¶^p = 0.045 vs controls

#### Tryptase positive mast cells (MC-T)

In central airways, the concentration of MC-T was highest in the subepithelial area in both groups (Table 2a). In peripheral airways (Figure 1), the concentration of MC-T was highest in the adventitia followed by the subepithelial area in both groups.

The concentration of MC-T was significantly lower in the subepithelial area of central airways from patients with COPD than from controls (53.7/mm^2 ^[25.7–120.8] versus 108.8/mm^2 ^[83.9–190.2], respectively, p = 0.04). Concentrations of MC-T in any of the other regions of central and peripheral airways were similar in both groups (p > 0.05).

MC-T numbers were significantly higher in subepithelial area in peripheral airways than in central airways in both COPD patients and controls (p = 0.01 and 0.03 respectively) without a difference in numbers in smooth muscle.

#### Chymase positive mast cells (MC-C)

Concentrations of MC-C were highest in the subepithelial area of central airways in both patients with COPD and controls (Table 2b). In peripheral airways, the concentration of MC-C was highest in the adventitia followed by the subepithelial area in both groups.

The concentration of MC-C was significantly higher in the epithelium of central airways from patients with COPD than from controls, but with very low cell numbers (0.0 [0.0–0.0]/mm^2 ^versus 0.0/mm^2 ^[0.0–0.3], respectively, p = 0.05). Concentrations of MC-C in any other of the regions of both central and peripheral airways were similar in both groups.

MC-C numbers were significantly higher in both subepithelial area and smooth muscle area of peripheral airways than in central airways in COPD patients (p = 0.03 and 0.05 respectively), but not in controls (p = 0.88 and 0.67 respectively).

The percentage of degranulated mast cells was higher in peripheral than in central airways for MC-T as well as MC-C (both p < .001), but was not significantly different between COPD and controls.

Exclusion of 2 control subjects with missing lung function data did not affect any of the results. A subgroup analysis excluding all COPD patients treated with corticosteroids provided similar results as presented above. The inter-group difference in subepithelial MC-T in central airways was reduced to a trend (p = 0.09), probably due to loss of power.

The ICC for the intra-observer variability was 0.96; the inter-observer variability for two independent observers resulted in an ICC of 0.98. This reflects excellent levels of agreement for repeat counts by either the same observer or two independent observers.

### Relation between mast cells and lung function in COPD patients

#### Tryptase positive mast cells

**Figure 2:**
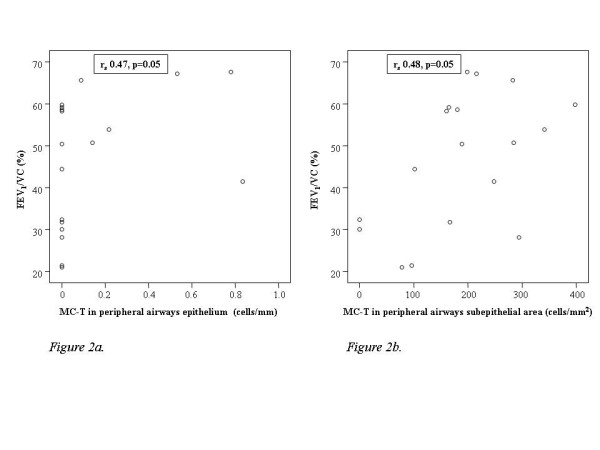
**The relation between the concentration of tryptase positive mast cells and lung function in patients with COPD.** 2a. MC-T in the epithelium of peripheral airways and FEV_1_/VC (%). 2b. MC-T in the subepithelial area of peripheral airways with FEV_1_/VC (%). FEV_1_/VC = the ratio of the forced expiratory volume in one second and vital capacity; MC-T = tryptase positive mast cells.

Higher numbers of MC-T in the epithelium and in the subepithelial area of peripheral airways correlated significantly with higher values of FEV_1_/VC (r_s _0.47, p = 0.05 and r_s _0.48, p = 0.05, respectively; figure 2a and 2b), but not with FEV_1_% pred (r_s _0.29, p = 0.25 and r_s _0.34, p = 0.17, respectively). Higher numbers of MC-T in the adventitia of peripheral airways tended to correlate with higher values of FEV_1_/VC (r_s _0.41, p = 0.09) and FEV_1_% pred (r_s _0.43, p = 0.08). A higher percentage of MC-T in the total airway wall area of peripheral airways also correlated significantly with higher values of FEV_1 _and FEV_1_/VC (r_s _0.52, p = 0.03 and r_s _0.55, p = 0.02).

No significant correlations existed between the number of MC-T in any of the other airway regions and lung function (either expressed as FEV_1_% pred or FEV_1_/VC).

#### Chymase positive mast cells

**Figure 3:**
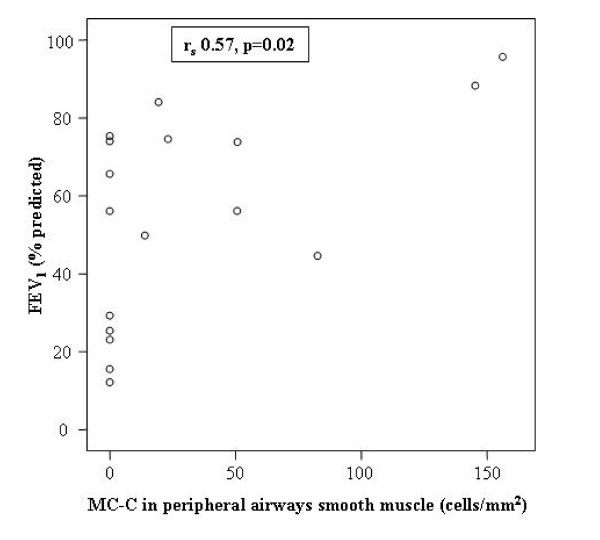
**The relation between the concentration of chymase positive mast cells in peripheral airways smooth muscle with FEV_1_% predicted in patients with COPD**. FEV_1 _= forced expiratory volume in one second; % predicted = percentage of the predicted value; MC-C = chymase positive mast cells.

Higher numbers of MC-C in the smooth airway muscle of peripheral airways correlated significantly with higher values of FEV_1_% pred (r_s_0.57, p = 0.02; figure 3), but not with FEV_1_/VC (r_s _0.35, p = 0.17). Higher numbers of MC-C in the adventitia of peripheral airways tended to correlate positively with FEV_1_% pred (r_s _0.43, p = 0.08).

We found no significant correlations between the number of MC-C in any of the other airway regions and lung function (either expressed as FEV_1_% pred or FEV_1_/VC).

## Discussion

In the present study, we demonstrate that *the distribution *of tryptase and chymase positive mast cells in the airways is similar in patients with COPD and controls without airflow limitation. In central airways, the concentration of both tryptase and chymase positive mast cells is highest in the subepithelial area. In peripheral airways, the concentration is highest in the adventitia, followed by almost similar high numbers in the subepithelial area. Interestingly, *the numbers *of mast cells differed between COPD and controls. Numbers of tryptase positive mast cells in the subepithelial area of central airways were lower in COPD than in controls, without other relevant significant differences. Finally, a higher number of tryptase and chymase positive mast cells in different regions and for tryptase also in the *total *wall area of peripheral airways is associated with less severe airflow limitation in COPD, relationships not observed in central airways.

Previous studies on the role of mast cells in COPD have not investigated both central and peripheral airways nor the relation of mast cells and their specific granule contents with lung function [5,16]. We observed no difference in the number of MC-T in peripheral airways between patients with COPD and controls. In contrast, Grashoff *et al*. demonstrated higher numbers of MC-T in the epithelium of peripheral airways, (but not in the remainder of the airway wall) in patients with COPD than in controls [5]. These seemingly incongruent findings may be due to the definition of COPD and controls used in both studies. We adhered to current guidelines [14,17] taking both FEV_1 _and FEV_1_/VC into account, whereas Grashoff's study, conducted before these guidelines were introduced, selected groups solely based on FEV_1_. Compatible with our findings, no correlation between MC-T in central airways and FEV_1 _was observed in another study investigating a heterogeneous population of 29 current smokers with and without airflow limitation [16]. The number of MC-T in peripheral airways was not assessed in the latter study. Interestingly, our findings are in keeping with a recent study in severe asthma [13], where higher numbers of MC-C in peripheral airways were associated with less severe airflow limitation [13]. However, in contrast to our findings, the asthma study did not observe such a relation for the number of MC-T and lung function. Thus, the present study is the first to report the relation of both tryptase and chymase positive mast cells in both central and peripheral airways with lung function in patients with COPD.

There are a few considerations when interpreting our results. Some studies [16] but not all [18], have shown higher numbers of airway mast cells in current than in ex- or never smokers. We observed a significantly lower number of MC-T in the subepithelial area of central airways in COPD than in controls. This might have been influenced by a higher percentage of current smokers in the control group, yet controls and COPD had similar smoking status. Furthermore, 13 of our 19 COPD patients were treated with corticosteroids versus none of the controls (p < 0.001). Treatment with corticosteroids can reduce mast cell numbers in central airways of patients with COPD [19,20]. However, as indicated in the results section, a subgroup analysis excluding all COPD patients treated with corticosteroids provided similar results as presented above.

Another point which might have had an impact on our results is that we used lung tissue obtained during surgery for lung cancer. The relation between tumour and mast cells has mainly been studied with respect to mast cells in the tumour tissue and/or effects of mast cells on the tumour [21]. In fact the only effects reported so far are related to mast cells in the immediate environment of tumour-cells or effects of chemotherapy on mast cell number and function.[21] To limit effects of the tumour on airflow limitation and inflammation, we only included patients, who had not received chemotherapy, with a peripherally localized tumour, and selected lung tissue as far as possible from the tumour. Taken together, we feel that the results we describe are valid.

How to put the present results in perspective of the underlying pathophysiology of COPD? The significant relation of MC-T and MC-C with airflow limitation in COPD was present in peripheral and not central airways, compatible with the widely accepted notion that peripheral airways are the major site of airflow limitation in COPD [2,22]. In line with a previous study in severe asthmatics [13], but yet to our surprise, a positive relation was found between mast cell numbers and lung function. This may either reflect a protective role of mast cells in the pathogenesis of COPD, or in contrast, it may represent a risk factor if lower numbers of MC-T and MC-C reflect increased degranulation of mast cells, leaving them undetected in an immunohistochemical approach [23]. Previous findings in asthma showed that the proportion of degranulated mast cells is higher in peripheral airways from patients with asthma than in controls [15]. In the present study, using the same protocol, we did not find a difference in degranulation in central or small airways between COPD patients and control subjects. For both groups we found more MC-T in peripheral than in subepithelial area of the central airways, whereas in COPD, but not in controls, there are less MC-C in the subepithelial area and more in the smooth muscle area in the peripheral airways than in the central airways. Moreover, for both groups we found a significantly higher percentage of MC-T and MC-C degranulation in peripheral than in central airways. Both numbers and percentage of degranulation of MC-T and MC-C were not different for COPD patients and control subjects. In the article and on-line supplement of Battaglia et al. [24] a similar increase of mast cells in peripheral airways has been described for ex- and current smokers, with or without COPD. Apparently, the quite extensive presence and degranulation of mast cells in peripheral airways does not necessarily lead to airway obstruction and may have other physiologic effects.

We realize that there are several arguments against a protective role for mast cells in COPD, since they secrete e.g. proteases, interleukin-8 [25] and tumour necrosis factor (TNF). [26,27] These cytokines contribute to neutrophilic inflammation in COPD [28-30], which is in turn associated with more severe airflow limitation [8]. Indeed mast cells generally have detrimental effects in various inflammatory conditions [31-33]. However, mast cells can have quite opposing effects in different situations, rather dependent on their micro-environment and the mast cell modulating factors that play a role at that particular microlocalization [9]. Moreover, there are potential beneficial effects as well. Mast cells were associated with a protective role in e.g. the development of glomerulonephritis in mice models of renal inflammatory disease [34]. Furthermore, they might protect against the detrimental smoke effects on lung function by the ability of tryptase to enhance epithelial cell proliferation [35], which could improve epithelial integrity. In addition, chymase may inhibit smooth muscle proliferation and thus the associated increased peripheral airway resistance in patients with COPD [36]. The proteases that are produced by mast-cells may limit the development of subepithelial fibrosis. Finally, mast cells can also have the opposite effect and contribute to fibroblast proliferation and collagen production [10-12,37]. This might increase extracellular matrix production in the adventitia, thereby counteracting effects of emphysematous destruction of peribronchial attachments.

In conclusion, in our view, the strength of the current study is that it is the first to investigate the distribution of tryptase and chymase positive mast cells in central and peripheral airways, and their relation with airflow limitation in patients with COPD. It remains to be clarified whether our findings reflect a beneficial or a detrimental effect of mast cells, or even a combination of both, on lung function in patients with COPD.

## Abbreviations

ASM: alpha-smooth-muscle actin; COPD: Chronic Obstructive Pulmonary Disease; FEV: Forced Expiratory Volume; GOLD: Global Initiative on Lung Disease; IQR: interquartile ranges; MC-C: mast cell-chymase; MC-T: mast cell-tryptase; VC: Vital Capacity.

## Competing interests

The authors declare that they have no competing interests.

## Authors' contributions

MG carried out the data analysis and drafted the manuscript. DP, NHTtH and WT participated in the design of the original study, were responsible for clinical and histological patient data and contributed substantially to the manuscript. BR, ML, MS, and MAL carried out the immunohistology experiments and contributed to the manuscript. JV assisted with data analysis and interpretation, supervised statistics and contributed to the manuscript.
